# A Novel H1N2 Influenza Virus Related to the Classical and Human Influenza Viruses from Pigs in Southern China

**DOI:** 10.3389/fmicb.2016.01068

**Published:** 2016-07-08

**Authors:** Yafen Song, Xiaowei Wu, Nianchen Wang, Guowen Ouyang, Nannan Qu, Jin Cui, Yan Qi, Ming Liao, Peirong Jiao

**Affiliations:** ^1^College of Veterinary Medicine, South China Agricultural UniversityGuangzhou, China; ^2^National and Regional Joint Engineering Laboratory for Medicament of Zoonosis Prevention and ControlGuangzhou, China; ^3^Key Laboratory of Animal Vaccine Development, Ministry of AgricultureGuangzhou, China; ^4^Key Laboratory of Zoonosis Prevention and Control of GuangdongGuangzhou, China; ^5^Guangdong Entry-Exit Inspection and Quarantine BureauGuangzhou, China; ^6^China Animal Husbandry GroupBeijing, China

**Keywords:** swine influenza virus, H1N2, reassortant, phylogenetic analysis, molecular characterization

## Abstract

Southern China has long been considered to be an epicenter of pandemic influenza viruses. The special environment, breeding mode, and lifestyle in southern China provides more chances for wild aquatic birds, domestic poultry, pigs, and humans to be in contact. This creates the opportunity for interspecies transmission and generation of new influenza viruses. In this study, we reported a novel reassortant H1N2 influenza virus from pigs in southern China. According to the phylogenetic trees and homology of the nucleotide sequence, the virus was confirmed to be a novel triple-reassortant H1N2 virus containing genes from classical swine (PB2, PB1, HA, NP, and NS genes), triple-reassortant swine (PA and M genes), and recent human (NA gene) lineages. It indicated that the novel reassortment virus among human and swine influenza viruses occurred in pigs in southern China. The isolation of the novel reassortant H1N2 influenza viruses provides further evidence that pigs are “mixing vessels,” and swine influenza virus surveillance in southern China will provide important information about genetic evaluation and antigenic variation of swine influenza virus to formulate the prevention and control measures for the viruses.

## Introduction

China is the biggest country for swine breeding and pork production, and the largest market for the consumption of pork in the world. It is also the only region that frequently imports pigs from other continents (Zhu et al., 2013; Kong et al., 2014). Swine influenza is an acute respiratory viral disease characterized by coughing, sneezing, nasal discharge, elevated rectal temperatures, lethargy, difficult breathing, and depressed appetite that decreases health and welfare of pigs and results in a significant economic loss for the swine industry worldwide (Kothalawala et al., 2006). As the RNA-dependent RNA polymerase could not proofread the newly synthesized gene segments, mutations of influenza A viruses arise in each replication cycle (Nichol et al., 2000; Koçer et al., 2013). It comes as no surprise; the viruses never stop changing and generating new viral subtypes via mutation, recombination and reassortant. Currently, H1N1, H3N2, and H1N2 swine influenza viruses (SIVs) are mainly circulating in the swine population in China, but H3N8, H4N8, H5N1, H6N6, and H9N2 influenza viruses have been also isolated in swine in China (Kong et al., 2014). Overall, special breeding environments and features of the virus result in various subtypes and genotype coexisting in China, which would accelerate the genomics evolutionary and antigenic variation of swine influenza viruses. Therefore, virologic and serologic surveillance of swine influenza virus is urgently required.

From January 2012 to March 2012, we carried out swine influenza virus surveillance in southern China. We collected 300 samples from pigs on swine farms in Guangdong province. One swine influenza virus was isolated. We analyzed the origin, genetic composition, and antigenicity characteristics of the hemagglutinin (HA) protein of the novel H1N2 subtypes isolated from pigs.

## Materials and methods

### Sample collection

From January 2012 to March 2012, we monitored swine influenza virus in swine farms in Guangdong province of southern China. We chose 30 swine farms and randomly collected 300 nasal swab samples from the 5 to 9-month-old fattening pigs, and also from sows, weaning pigs, nursery pigs, and boars which showed suspicious clinical symptoms. The samples were sent to our laboratory, and stored at −80°C until analysis. This study of nasal sampling from pigs was carried out in accordance with the recommendations of the experimental animal administration and ethics committee of South China Agriculture University of guidelines. The protocol was approved by the biosafety committee of South China Agriculture University.

### Virus isolation and identification

The collected samples were inoculated into amnionic and allantoic cavities of 9–10-day-old specific-pathogen-free (SPF) embryonated chickens eggs. After incubating for 48 h at 37°C, the allantoic fluids were harvested, and the reverse-transcription polymerase-chain reaction (RT-PCR), hemagglutination test, and hemagglutination inhibition (HI) test were performed to identify and subtype the positive influenza samples as described previously (Song et al., 2015). Finally, virus allantoic fluids were harvested and stored at −80°C before use. All experiments were carried out in ABSL-3 facilities in compliance with the biosafety committee of South China Agriculture University protocols. All experiments handling was performed in accordance with the experimental animal administration and ethics committee of South China Agriculture University guidelines.

### Gene sequence and molecular analysis

Viral RNA was extracted from allantoic fluid with Trizol LS Reagent (Life Technologies, Inc.) and transcribed into cDNA with SuperScript III reverse transcriptase (Invitrogen, China). PCR was performed as described previously (Song et al., 2015). The products were purified with the QIAquick PCR purification kit (QIAGEN) following the manufacturer's instructions and sequencing was performed by using an ABI Prism 3730 genetic analyzer (Applied Biosystems) by Shanghai Invitrogen Biotechnology Co., Ltd.

Sequencing data were compiled with the SEQMAN program of Lasergene 7 (DNASTAR). All the referred sequences of this article were downloaded from NCBI databases. BLAST analysis was carried out on NCBI. The consensus sequences of each lineage were obtained using MegAlign and then compared with MEGA (version 4.0) using Clustal W Method. Phylogenetic trees were generated with MEGA program (version 4.0) using neighbor-joining analysis. Bootstrap value was calculated on 1000 replicates of the alignment. The nucleotide sequences in this study are available on GenBank (accession number KX269879-KX269886).

## Results

### Virus isolation and identification

The virus was isolated from nasal swab samples of nursery pigs, and identified by the reverse-transcription polymerase-chain reaction (RT-PCR), hemagglutination test, and hemagglutination inhibition (HI) test and confirmed by genomic sequencing and the nucleotide BLASTn analysis. According to the results, the virus was identified as swine influenza A (H1N2) virus and named as A/swine/Guangdong/1/2012(H1N2).

### Homology analysis of nucleotide sequences

To understand whether the swine H1N2 isolate [A/swine/Guangdong/1/2012(H1N2)] is related to the previous swine H1N2 viruses, eight gene segments of the virus were sequenced, and the homology was determined by comparison with the sequences available in GenBank. Viral homology analysis of nucleotide sequences of the virus was presented in Table 1 and Figure 9. The PB2 gene of the virus shared the highest nucleotide sequence identity with a triple-reassortant swine (TRIG) H1N2 influenza virus (Vijaykrishna et al., 2011), A/swine/Hong Kong/NS30/2004 (H1N2), with a homology rate of 97.5%. The PB1 shared the highest nucleotide sequence identity with another TRIG H1N2 influenza virus [A/swine/Hong Kong/NS1890/2009 (H1N2)], with a homology rate 96.9%. The PA and NP genes both shared the highest nucleotide sequence identity with A/swine/Hong Kong/1111/2004 (H1N2), which was another TRIG H1N2 influenza virus isolated in Hong Kong, with a homology rate ranging from 97.1 to 97.6%. The HA gene was the most closely related to the classical swine (CS) H1N2 viruses (A/swine/Hainan/1/2005 (H1N2; Yu et al., 2009), with a homology rate 96.8%. The NA gene showed a close relationship with A/swine/Hong Kong/294/2009 (H1N2; with a homology rate 97.1%), which was a TRIG H1N2 influenza virus that acquired the HA gene from the CS viruses (Smith et al., 2009). The M and NS genes shared the highest nucleotide sequence identity with another TRIG H1N2 influenza virus (Smith et al., 2009), A/swine/Hong Kong/NS623/2002 (H1N2), with a homology rate ranging from 97.1 to 97.9%. Thus, the results of the homology analysis suggested that the virus might be a multi-reassortant virus.

**Table 1:** **Nucleotide sequence identity between the isolate virus and reference strains available in GenBank^**a**^**.

**Gene^b^**	**Virus with the greatest similarity**	**Identity (%)**
HA	A/swine/Hainan/1/2005(H1N2)	96.8
NA	A/swine/Hong Kong/294/2009(H1N2)	97.1
PB2	A/swine/Hong Kong/NS30/2004(H1N2)	97.5
PB1	A/swine/Hong Kong/NS1890/2009(H1N2)	96.9
PA	A/swine/Hong Kong/1111/2004(H1N2)	97.6
NP	A/swine/Hong Kong/1111/2004(H1N2)	97.1
M	A/swine/Hong Kong/NS623/2002(H1N2)	97.9
NS	A/swine/Hong Kong/NS623/2002(H1N2)	97.1

a *http://www.ncbi.nlm.nih.gov/*.

b *HA, hemagglutinin; NA, neuraminidase; PB, polymerase basic subunit; PA, polymerase acidic subunit; NP, nucleoprotein; M, matrix; NS, nonstructural*.

### Phylogenetic analysis of the virus

To understand the genetic origin of the gene segments of the virus more precisely, eight phylogenetic trees were constructed using the nucleotide sequences of the virus and the genes of reference viruses available in GenBank, which included viruses isolated from poultry, human, and swine.

**Figure 1:**
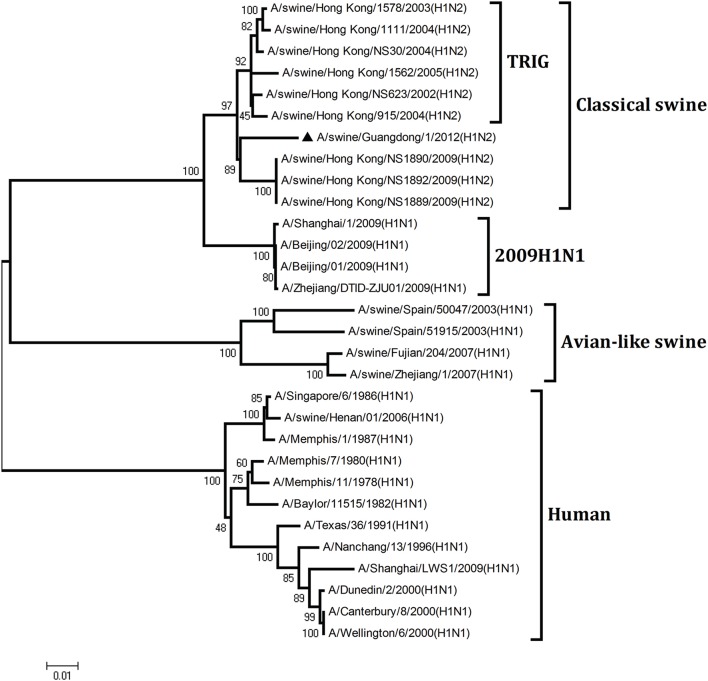
**Continued**.

**Figure 2:**
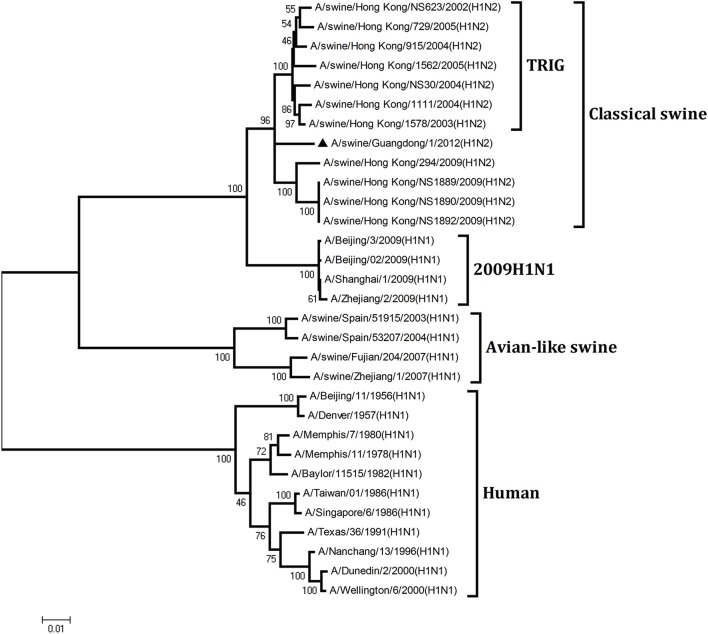
**Continued**.

**Figure 3:**
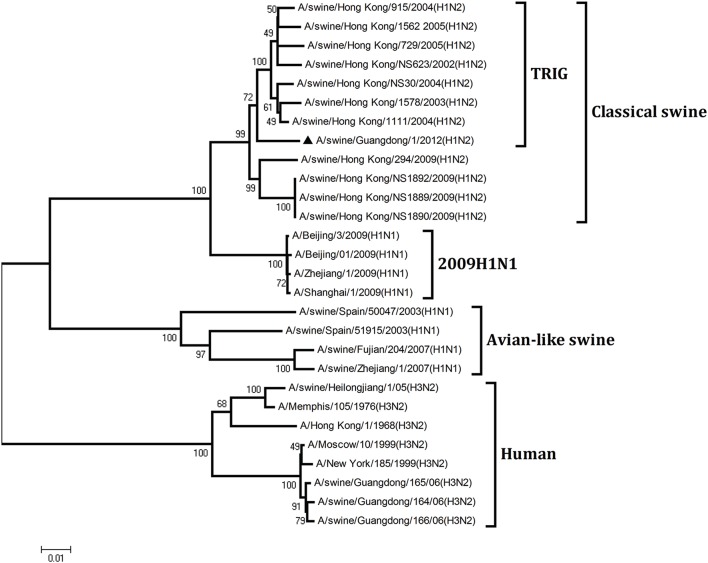
**Continued**.

**Figure 4:**
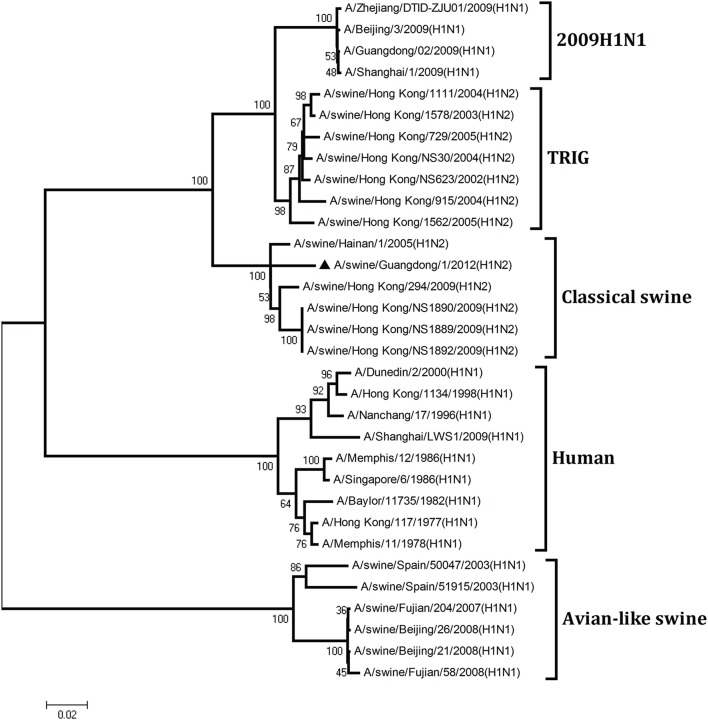
**Continued**.

**Figure 5:**
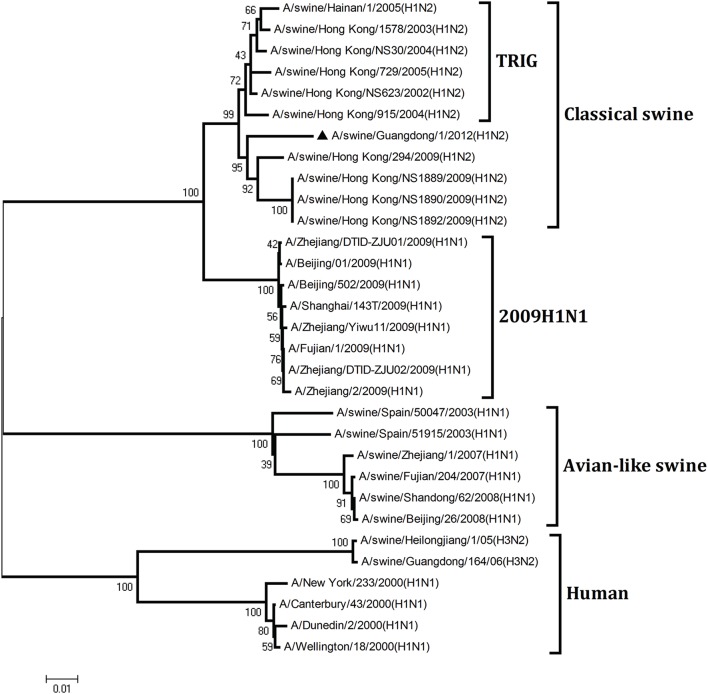
**Continued**.

**Figure 6:**
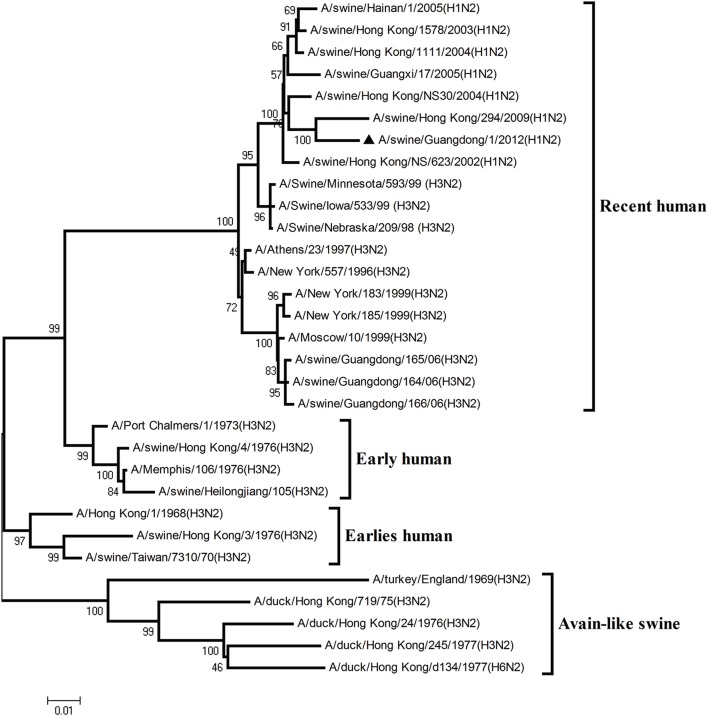
**Continued**.

**Figure 7:**
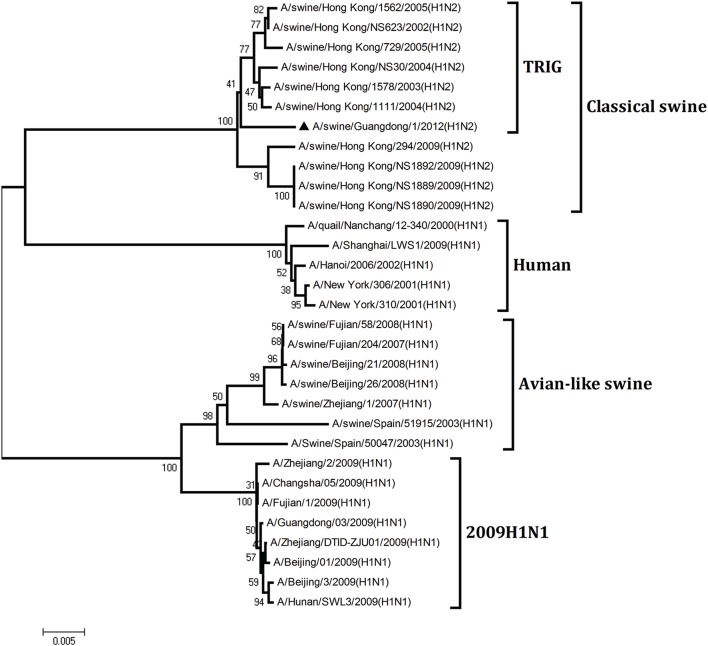
**Continued**.

**Figure 8:**
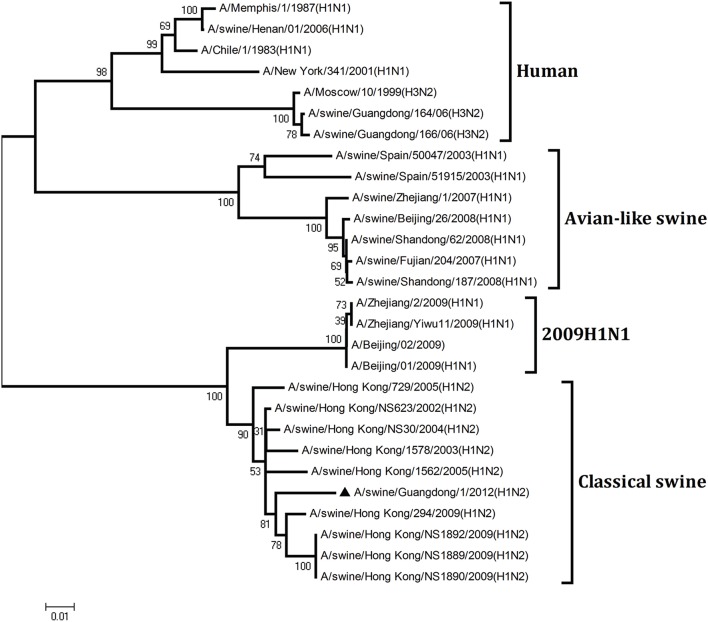
**Phylogenetic trees of the PB2, PB1, PA, HA, NP, NA, M, and NS genes of influenza A viruses**. The virus marked with “▴” is the swine H1N2 virus [A/swine/Guangdong/1/2012 (H1N2)] isolated and sequenced in this study. The unrooted phylogenetic trees were generated by the distance-based neighbor-joining method using MEGA 4.0 software based on the following sequences: PB2 gene (1), nt 1–2280; PB1 gene (2), nt 1–2274; PA gene (3), nt 1–2151; HA gene (4), nucleotides (nt) 33–1733; NP gene (5), nt 46–1542; NA gene (6), nt 20–1429; M1 (7) gene, nt 11–982; NS1 gene (8), nt 1–660. Bootstrap value was calculated on 1000 replicates of the alignment.

The phylogenetic analysis results showed that the PB2, PB1, HA, NP, and NS genes of the novel H1N2 virus all fell into the classical swine lineage (Figures 1, 2, 4, 5, 8). The PA and M genes of the virus belonged to the TRIG lineage (Figures 3, 7). The NA gene of A/swine/Guangdong/1/2012(H1N2) was special and segregated into recent human lineage, early human lineage, earliest human lineage, and avian-like swine lineage (Figure 6). Though the NA gene of the virus was closely related to that of those TRIG H1N2 influenza viruses, it fell into the recent human lineage, which containing human H3N2 influenza viruses isolated from the 1990s and the Twenty-first century. Therefore, according to the phylogenetic trees and homology of the nucleotide sequence, the virus was confirmed to be a novel triple-reassortant H1N2 virus containing genes from classical swine (PB2, PB1, HA, NP, and NS genes), TRIG (PA and M genes), and recent human (NA gene) lineages.

**Figure 9:**
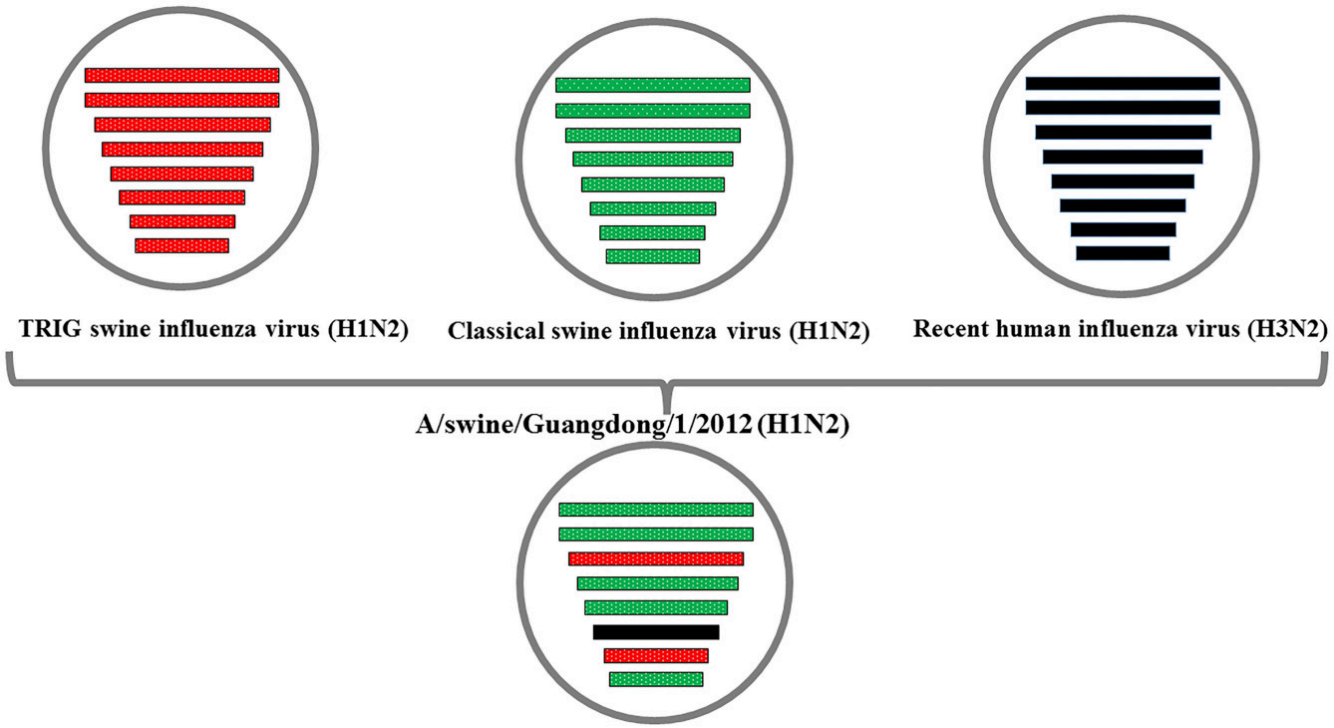
**Putative genomic compositions of the novel swine influenza (H1N2) virus (A/swine/Guangdong/1/2012)**. The eight genes of these viruses were represented by horizontal bars. From top to bottom, PB2, PB1, PA, HA, NP, NA, M, and NS are indicated. Each different color represents a distinct origin.

### Molecular analysis

**Figure 10:**
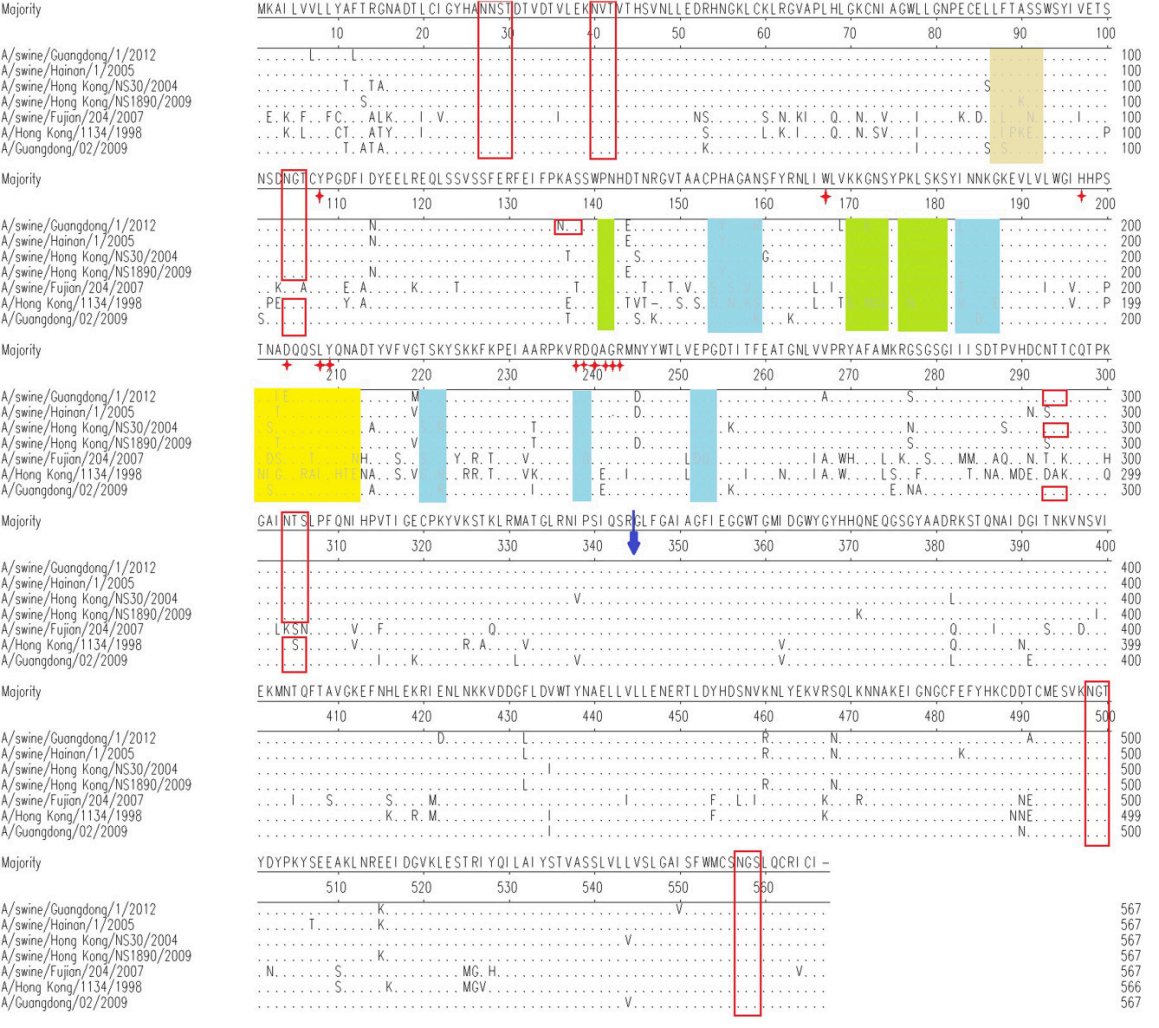
**Molecular analysis of HA amino acid sequences of the isolated virus and reference strains**. Red boxes are potential glycosylation sites. Previously defined antigenic sites are indicated: site Sa (lime shade), site Sb (yellow shade), site Ca (pale green shade), site Cb (canary shade). Residues marked with “
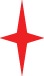
” are receptor-binding sites. The site with a blue arrow is the HA cleavage site.

In our study, the deduced amino acid sequences of the HA region of the novel H1N2 virus and other representative influenza viruses from China were aligned and analyzed. The novel H1N2 virus and other representative viruses all contained an amino acid motif PSIQSR↓G at their HA cleavage sites, which is a characteristic of low pathogenic influenza viruses. Five potential glycosylation sites (N–X–S/T) were conserved at positions 27, 28, 40, 498, and 557 (H1 numbering) in the HA protein of all analyzed viruses (Figure 10). The viruses from human, classical swine, and 2009H1N1 lineages also had conserved glycosylation sites at positions 104 and 304 (H1 numbering). One potential glycosylation sites at position 293 (H1 numbering) only existed in some viruses. The K136N (H1 numbering) mutation brought a new potential glycosylation site at position 136 in the HA protein of the novel H1N2 virus. In addition, the viruses of the human, classical swine, and 2009H1N1 lineages had more glycosylation sites than those of the avian-like lineage. The novel H1N2 virus had the same D at positions 225 of HA as the 1918 human strain, suggesting that the virus may have potential to infect humans. Antigenic sites are regions of molecules involved in antibody binding. Several mutations were found in the antigenic sites of the A/swine/Guangdong/1/2012(H1N2) virus (Figure 10): G158E at site Sa; S188N, A189I, and D204E at site Sb; S140P, H141Y, N145R, I169L, S206T, K224R, and Q240G at site Ca; and S74F and N77S at site Cb (H3 numbering).

In our study, we found five glycosylation sites in the NA protein in the novel H1N2 virus. Two were in the linker region (N61 and N70) and the other three were in the NA domain (N146, N200, and N234). The amino acid substitutions (E119G, H274Y, R292K, and N294S) were not observed in the NA protein of the novel H1N2 virus, which suggested the virus was still sensitive to NA inhibitors.

Analysis based on the deduced amino acid sequence of A/swine/Guangdong/1/2012(H1N2) and its potential donor virus, the novel H1N2 virus and its potential donor virus [A/swine/Hong Kong/NS30/2004 (H1N2)] both contained 271A, 590S, 591R, 627E, and 701D in the PB2 protein. The isolate and its potential donor virus [A/Hong Kong/NS1890/2009(H1N2)] both contained a truncated PB1-F2 protein (57aa) and a PB1-N40 protein (718aa). The PA-X protein of swine influenza viruses usually exists in either full length or a truncated form (either 61aa or 41aa). The novel H1N2 virus and A/Hong Kong/1111/2004 (H1N2) both possessed a full-length PA-X. The amino acid substitutions (26, 30, 31, and 34) were not observed in the M2 protein of the novel H1N2 virus and its potential donor virus A/swine/Hong Kong/NS623/2002(H1N2). But V27I substitution both occurred in the M2 protein of them.

## Discussion

Swine influenza was first observed as a pertinent disease of swine in 1918 at the time of the human pandemic, and the virus was isolated and identified in 1930. This is known as the “classical” H1N1 swine virus. From 1918 to 1919, the classical swine influenza virus caused a high mortality among pigs in Chinese coastal cities. The classical swine virus was first isolated in Hong Kong of China in 1974 and continued to presence in apparently healthy pigs in Hong Kong and Mainland China (Zhu et al., 2013). Recently, classical H1N1 swine virus emerged in humans as a reassortant (2009/H1N1) and caused the 2009 H1N1 influenza pandemic (Dawood et al., 2009). Reassortant H1N2 influenza A viruses derived from human-like swine H3N2 and classical swine H1N1 viruses were first isolated in Japan in 1978 and became endemic in Japanese swine populations (Sugimura et al., 1980). Then, these reassortant swine H1N2 influenza viruses have also been isolated and demonstrated in many countries, such as France, the United Kingdom, the United States, Korea, Spain, Germany, Thailand, and so on (Yu et al., 2009). However, these reassortant H1N2 viruses were not reported until 2004 in China (Qi and Lu, 2006).

In our study, a reassortant swine influenza virus (A/swine/Guangdong/1/2012) was isolated from nasal swab samples of nursery pigs when we collected a total of 300 samples from pigs in swine farms of Guangdong province during swine influenza virus surveillance between January 2012 and March 2012. Homology analysis showed that the HA gene was most closely related to the classical swine (CS) H1N2 viruses and the NA gene showed a closer relationship with a TRIG H1N2 influenza virus that acquired the HA gene from the CS viruses. Phylogenetic analysis showed that the PB2, PB1, HA, NP, and NS genes of the virus fell into the classical swine lineage, and the NA gene belonged to the recent human lineage. The PA and M genes of the virus belonged to TRIG lineage. The data confirmed that A/swine/Guangdong/1/2012(H1N1) was a novel triple-reassortant H1N2 virus, and the recombination event occurred in swine populations of southern China.

In general, species barriers prevent the movement of influenza viruses from one host into another host. For complete adaptation and further transmission between species, influenza A virus must overcome species barriers including spatial, physiological, and molecular in origin (Koçer et al., 2013). When influenza A virus shed by one host to infect another, it must breach entry barriers. The specificity of receptor molecules usually governs viral entry into cells. It is well-known that avian influenza viruses preferentially bind to sialic acids (SA)-α-2,3-Gal–terminated glycoproteins, whereas human influenza viruses bind SA-α-2,6-Gal–terminated glycoproteins. In humans, epithelial cells of the upper airway predominantly express SA-α-2,6-Gal–terminated glycoproteins. These differences may be responsible for restricting replication of avian viruses in humans. However, respiratory epithelia of pigs express both SA-α-2,3-Gal–terminated glycoproteins and SA-α-2,6-Gal–terminated glycoproteins. Thus, pigs are susceptible to infection with both avian and human influenza virus suggesting that pigs are a potential source for generating novel reassortant influenza viruses and are more frequently involved in interspecies transmission of influenza A viruses than other animals. Therefore, pigs are always considered “mixing vessels” (Brown, 2001; Kuiken et al., 2006). The outbreak of the swine-origin triple-reassortant H1N1 influenza virus in 2009, which contained the genes from human, avian, and swine influenza viruses, is a representative example of emerging viruses that are recombined and adapted in pigs before transmission to humans (Smith et al., 2009). In our study, the isolate had the same D at positions 225 of the receptor-binding property of HA protein as the 1918 human strain, suggesting that the virus may have potential to infect humans.

The antigenic evolution of the influenza virus via genetic processes of antigenic drift and shift increases antigen variably and leads to epidemics and pandemics. Antigenic drift usually occurs in the antibody-binding sites in the HA protein, the NA protein, or both. It is responsible for the selection pressure to evade host immunity. Lacking immunity in the newly drifted virus will result in a more severe, early-onset influenza epidemic, and increased mortality (Zambon, 2001; Carrat and Flahault, 2007). In our results, we found some changes in the antigenic sites of the A/swine/Guangdong/2012 virus: G158E at site Sa; S188N, A189I, and D204E at site Sb; S140P, H141Y, N145R, I169L, S206T, K224R, and Q240G at site Ca; and S74F and N77S at site Cb (H3 numbering). More studies should be done to determine whether these changes in antigenic sites would prompt the reassorted swine virus to infect swine and other hosts.

The amino acid at 627 of PB2 is a key factor for host range, and all human influenza viruses (H1N1, H2N2, and H3N2) have K at this position, whereas the majority of avian influenza viruses have E (Qi et al., 2009). 271A with the 590/591 SR polymorphism in PB2 protein helps pH1N1 and triple-reassortant swine influenza viruses overcome host restriction and efficient replication and adaptation in mammals (Liu et al., 2012). When avian-like signature 627E remains stable rather than changing to the mammalian-like signature 627K, a compensatory D701N substitution increased the polymerase activity and enhanced virulence in mice and enhanced transmission between guinea pigs (Li et al., 2006; Zhou et al., 2013). In our study, the A/swine/Guangdong/1/2012 contained 271A, 590S, 591R, 627E, and 701D in the PB2 protein. The 271A, 590S, and 591R may help our isolate overcome host restriction and efficient replication and adaptation in mammals. PB1-F2 is encoded in an alternative reading frame of the PB1 gene and a small protein which is transported to the mitochondria and nucleus. PB1-F2 could induce apoptosis in the host cell and increase virulence and the risk of secondary infections (Chen et al., 2001; McAuley et al., 2007). PB1-F2 protein has variable sizes with truncations either at the C- or N-terminal ends (Vasin et al., 2014). Studies demonstrated that H5N1influenza A virus containing a PB1-F2 was more virulent for BALB/c mice than a closely related H5N1 containing intact PB1-F2 (Kamal et al., 2015). However, a truncated PB1-F2 did not affect the pathogenesis of H1N1 seasonal influenza virus (Meunier and von Messling, 2012). In our study, the isolate and its potential donor virus both contained a truncated PB1-F2 protein (57aa), the role of truncated PB1-F2 of them should be studied in the future. PA-X protein is expressed from a second open reading frame of the PA gene. Studies demonstrated that PA-X decreased the pathogenicity of pandemic 1918 H1N1 virus, 2009 pandemic H1N1 (pH1N1), and highly pathogenic avian influenza H5N1 viruses in mice by modulating the host response (Jagger et al., 2012; Koçer et al., 2013; Gao et al., 2015; Hu et al., 2015). However, Gao et al. demonstrated that PA-X protein in H9N2 virus was a pro-virulence factor in facilitating viral pathogenicity (Gao et al., 2015). In our study, the novel H1N2 and its potential donor virus both possessed a full-length PA-X. The pro- or anti-virulence role of PA-X of them should be studied in the future.

The prevention and control of influenza virus mainly relies on antiviral drugs and vaccines. Amantadine, an adamantane derivative, is an antiviral compound effective against influenza virus. Despite certain side-effects and a rapid induction of resistant strains, amantadine is licensed for the prophylaxis and therapy of influenza in various countries. It inhibits the function of the influenza virus M2 proton channel and single amino acid substitutions at positions L26F, V27A(T), A30T, S31N, and G34E of the M2 protein to confer resistance against it. Single mutant with S31N or double mutants with the S31N and either of the L26F, V27A, or V27T substitutions both confers amantadine resistance (Abed et al., 2005; Krumbholz et al., 2009), However, the significance of V27I exchanges need further study. On the other hand, oseltamivir is used as an NA inhibitor in the treatment of infecting influenza H5N1viruses. Moreover, if the substitutions E119E, H274Y, R292K, and N284S in the NA protein happen, the influenza virus may not be sensitive to the NA inhibitors. In our study, the isolate was still sensitive to NA inhibitors.

Although swine influenza is widespread and is endemic throughout the world, isolating swine influenza viruses is relatively difficult and is dependent on the time of sampling. And the continual co-circulation of antigenically diverse swine influenza virus is a challenge to the production of efficacious and protective vaccines. Antigen-specific antibodies induced by current vaccines provide limited cross protection to heterologous challenges. Moreover, many studies have demonstrated that the vaccine is correlated with an increased risk of influenza-like-illness in swine. Thus, this is why the vaccine is rarely used in swine populations in China. Therefore, it is important to develop new vaccines with high efficacy and safety to protect the swine from influenza viruses in the future.

## Author contributions

Conceived and designed the experiments: PJ. Performed the experiments: YS, XW. Analyzed the data: PJ, YS, XW. Contributed reagents/materials/analysis tools: PJ, XW, YS, NW, GO, NQ, JC. Wrote the paper: YS, YQ, PJ. All authors read and approved the final manuscript.

### Conflict of interest statement

The authors declare that the research was conducted in the absence of any commercial or financial relationships that could be construed as a potential conflict of interest.

## References

[B1] Abed Y. Goyette N. Boivin G. (2005). Generation and characterization of recombinant influenza A (H1N1) viruses harboring amantadine resistance mutations. Antimicrob. Agents Chemother. 49, 556–559. 10.1128/AAC.49.2.556-559.2005 15673732 PMC547263

[B2] Brown I. H. (2001). The pig as an intermediate host for influenza A viruses between birds and humans, in Options for the Control of Influenza Iv (Crete) 1219, 173–178.

[B3] Carrat F. Flahault A. (2007). Influenza vaccine: the challenge of antigenic drift. Vaccine 25, 6852–6862. 10.1016/j.vaccine.2007.07.027 17719149

[B4] Chen W. Calvo P. A. Malide D. Gibbs J. Schubert U. Bacik I.. (2001). A novel influenza A virus mitochondrial protein that induces cell death. Nat. Med. 7, 1306–1312. 10.1038/nm1201-1306 11726970

[B5] Dawood F. S. Jain S. Finelli L. Shaw M. W. Lindstrom S. Garten R. J.. (2009). Emergence of a novel swine-origin influenza A (H1N1) virus in humans. N. Engl. J. Med. 360, 2605–2615. 10.1056/NEJMoa0903810 19423869

[B6] Gao H. Sun Y. Hu J. Qi L. Wang J. Xiong X.. (2015). The contribution of PA-X to the virulence of pandemic 2009 H1N1 and highly pathogenic H5N1 avian influenza viruses. Sci. Rep. 5:8262. 10.1038/srep08262 25652161 PMC4317690

[B7] Hu J. Mo Y. Wang X. Gu M. Hu Z. Zhong L.. (2015). PA-X decreases the pathogenicity of highly pathogenic H5N1 influenza A virus in avian species by inhibiting virus replication and host response. J. Virol. 89, 4126–4142. 10.1128/JVI.02132-14 25631083 PMC4442343

[B8] Jagger B. W. Wise H. M. Kash J. C. Walters K. A. Wills N. M. Xiao Y. L.. (2012). An overlapping protein-coding region in influenza A virus segment 3 modulates the host response. Science 337, 199–204. 10.1126/science.1222213 22745253 PMC3552242

[B9] Kamal R. P. Kumar A. Davis C. T. Tzeng W. P. Nguyen T. Donis R. O.. (2015). Emergence of highly pathogenic avian influenza A(H5N1) virus PB1-F2 variants and their virulence in BALB/c mice. J. Virol. 89, 5835–5846. 10.1128/JVI.03137-14 25787281 PMC4442455

[B10] Koçer Z. A. Jones J. C. Webster R. G. (2013). Emergence of influenza viruses and crossing the species barrier. Microbiol. Spectr. 10.1128/microbiolspec.OH-0010-2012 26184958

[B11] Kong W. Ye J. Guan S. Liu J. Pu J. (2014). Epidemic status of Swine influenza virus in china. Indian J. Microbiol. 54, 3–11. 10.1007/s12088-013-0419-7 24426160 PMC3889855

[B12] Kothalawala H. Toussaint M. J. Gruys E. (2006). An overview of swine influenza. Vet. Q. 28, 46–53. 10.1080/01652176.2006.9695207 16841566

[B13] Krumbholz A. Schmidtke M. Bergmann S. Motzke S. Bauer K. Stech J.. (2009). High prevalence of amantadine resistance among circulating European porcine influenza A viruses. J. Gen. Virol. 90, 900–908. 10.1099/vir.2008.007260-0 19223487

[B14] Kuiken T. Holmes E. C. McCauley J. Rimmelzwaan G. F. Williams C. S. Grenfell B. T. (2006). Host species barriers to influenza virus infections. Science 312, 394–397. 10.1126/science.1122818 16627737

[B15] Li Z. Jiang Y. Jiao P. Wang A. Zhao F. Tian G.. (2006). The NS1 gene contributes to the virulence of H5N1 avian influenza viruses. J. Virol. 80, 11115–11123. 10.1128/JVI.00993-06 16971424 PMC1642184

[B16] Liu Q. Qiao C. Marjuki H. Bawa B. Ma J. Guillossou S.. (2012). Combination of PB2 271A and SR polymorphism at positions 590/591 is critical for viral replication and virulence of swine influenza virus in cultured cells and *in vivo*. J. Virol. 86, 1233–1237. 10.1128/JVI.05699-11 22072752 PMC3255826

[B17] McAuley J. L. Hornung F. Boyd K. L. Smith A. M. McKeon R. Bennink J.. (2007). Expression of the 1918 influenza A virus PB1-F2 enhances the pathogenesis of viral and secondary bacterial pneumonia. Cell Host Microbe 2, 240–249. 10.1016/j.chom.2007.09.001 18005742 PMC2083255

[B18] Meunier I. von Messling V. (2012). PB1-F2 modulates early host responses but does not affect the pathogenesis of H1N1 seasonal influenza virus. J. Virol. 86, 4271–4278. 10.1128/JVI.07243-11 22318139 PMC3318652

[B19] Nichol S. T. Arikawa J. Kawaoka Y. (2000). Emerging viral diseases. Proc. Natl. Acad. Sci. U.S.A. 97, 12411–12412. 10.1073/pnas.210382297 11035785 PMC34064

[B20] Qi X. Lu C. P. (2006). Genetic characterization of novel reassortant H1N2 influenza A viruses isolated from pigs in southeastern China. Arch. Virol. 151, 2289–2299. 10.1007/s00705-006-0796-x 16755371 PMC7087176

[B21] Qi X. Pang B. Lu C. P. (2009). Genetic characterization of H1N1 swine influenza A viruses isolated in eastern China. Virus Genes 39, 193–199. 10.1007/s11262-009-0375-9 19521758

[B22] Smith G. J. Vijaykrishna D. Bahl J. Lycett S. J. Worobey M. Pybus O. G.. (2009). Origins and evolutionary genomics of the 2009 swine-origin H1N1 influenza A epidemic. Nature 459, 1122–1125. 10.1038/nature08182 19516283

[B23] Song Y. Cui J. Song H. Ye J. Zhao Z. Wu S.. (2015). New reassortant H5N8 highly pathogenic avian influenza virus from waterfowl in Southern China. Front. Microbiol. 6:1170. 10.3389/fmicb.2015.01170 26557113 PMC4615950

[B24] Sugimura T. Yonemochi H. Ogawa T. Tanaka Y. Kumagai T. (1980). Isolation of a recombinant influenza virus (Hsw 1 N2) from swine in Japan. Arch. Virol. 66, 271–274. 10.1007/BF01314741 6255900

[B25] Vasin A. V. Temkina O. A. Egorov V. V. Klotchenko S. A. Plotnikova M. A. Kiselev O. I. (2014). Molecular mechanisms enhancing the proteome of influenza A viruses: an overview of recently discovered proteins. Virus Res. 185, 53–63. 10.1016/j.virusres.2014.03.015 24675275

[B26] Vijaykrishna D. Smith G. J. Pybus O. G. Zhu H. Bhatt S. Poon L. L.. (2011). Long-term evolution and transmission dynamics of swine influenza A virus. Nature 473, 519–522. 10.1038/nature10004 21614079

[B27] Yu H. Zhang P. C. Zhou Y. J. Li G. X. Pan J. Yan L. P.. (2009). Isolation and genetic characterization of avian-like H1N1 and novel ressortant H1N2 influenza viruses from pigs in China. Biochem. Biophys. Res. Commun. 386, 278–283. 10.1016/j.bbrc.2009.05.056 19460353

[B28] Zambon M. C. (2001). The pathogenesis of influenza in humans. Rev. Med. Virol. 11, 227–241. 10.1002/rmv.319 11479929

[B29] Zhou B. Pearce M. B. Li Y. Wang J. Mason R. J. Tumpey T. M.. (2013). Asparagine substitution at PB2 residue 701 enhances the replication, pathogenicity, and transmission of the 2009 pandemic H1N1 influenza A virus. PLoS ONE 8:e67616. 10.1371/journal.pone.0067616 23799150 PMC3683066

[B30] Zhu H. Webby R. Lam T. T. Smith D. K. Peiris J. S. Guan Y. (2013). History of Swine influenza viruses in Asia. Curr. Top. Microbiol. Immunol. 370, 57–68. 10.1007/82_2011_179 21948002

